# Molecular dynamics study on the relationship between phase transition mechanism and loading direction of AZ31

**DOI:** 10.1038/s41598-021-96469-3

**Published:** 2021-08-26

**Authors:** Qianhua Yang, Chun Xue, Zhibing Chu, Yugui Li, Lifeng Ma, Hong Gao

**Affiliations:** 1 College of Materials Science and Engineering, Taiyuan University of Science and Technology, Taiyuan, 030024 China; 2 College of Mechanical Engineering, Taiyuan University of Science and Technology, Taiyuan, 030024 China; 3 Jiangsu Wujin Stainless Steel Co., Ltd, Changzhou, 213000 China

**Keywords:** Materials science, Nanoscience and technology

## Abstract

To develop and design mg-based nanoalloys with excellent properties, it is necessary to explore the forming process. In this paper, to explore the effect of different loading directions on the phase transformation of magnesium alloy, the model of AZ31 magnesium alloy was established, the process of Uniaxial Compression (UC) of magnesium alloy in different directions was simulated, the changes of atomic position and phase structure were observed, and the phase transformation mechanism of AZ31 magnesium alloy under uniaxial compression under different loading directions was summarized. The conclusions are as follows: the stress and strain, potential energy and volume change, void evolution, phase structure change and dislocation evolution of magnesium alloy are consistent, and there is no significant difference. In the process of uniaxial compression, the phase transformation of hexagonal closely packed (HCP) → face-centered cubic (FCC) is the main, and its structure evolves into HCP → Other → FCC. Shockley partial dislocations always precede FCC stacking faults by about 4.5%, and Shockley partial dislocations surround FCC stacking faults. In this paper, the phase transformation mechanism of AZ31 magnesium alloy under uniaxial compression under different loading directions is summarized, which provides a theoretical basis for the processing and development of magnesium-based nanoalloys.

## Introduction

Magnesium alloys are commonly used in the aerospace and automotive industry because of their low density and excellent mechanical properties^1–8^. However, magnesium alloy shows obvious anisotropy during deformation at room temperature, because its hexagonal closely packed (HCP) structure has less slip system^9^. Nanocrystalline metals and alloys will show different characteristic mechanical properties from coarse-grained metals and alloys. Compared with coarse-grained polycrystalline materials, nanocrystalline materials show higher strength, hardness, and toughness. Nanostructure will be an effective way to improve the strength and plasticity of magnesium alloys in the future^10–16^. Therefore, it is more and more necessary to study the changes of microstructure and mechanical properties in the process of material processing from the basic process of molecular motion^17^. With the progress of science and technology, computer simulation is widely used to explore the microstructure and mechanical properties of materials, and the typical application is molecular dynamics simulation (MD)^18–23^. as a new means of calculating materials, molecular dynamics simulation has been successfully used to study lattice distortion, grain deformation, and so on^24^.

Sunil Rawat^25^ studied the plasticity (twin and dislocation slip) and ω phase transition of single crystal titanium under different strain directions by molecular dynamics simulation. The loading perpendicular to the c axis leads to the activation and ω phase transition of $\left\{ {10\overline{1}2} \right\}$ twins. For loading in the $[2\overline{1 }\overline{1 }0]$ direction, four twin variables (two conjugate pairs) are activated, and for loading in the $[01\overline{1 }0]$ direction, two twin variables (one conjugate pair) are activated. Xiaoqin Ou^26^ uniaxial tensile tests of nano-face-centered cubic iron were carried out along $[\overline{1 }\overline{1 }2]$, $[1\overline{1 }0]$ and $[111]$, respectively, through molecular dynamics simulation. The applied tension along with the $[\overline{1 }\overline{1 }2]$ direction leads to the transformation of the body-centered cube in the face-centered vertical direction. The stable cubic core of flat ellipsoid is composed of $(01\overline{1 })$ body-centered cubic twin martensite structure. The stretching direction is $[1\overline{1 }0]$, and the two groups of plate-centered cubic fibers perpendicular to each other form a face-centered cubic phase with the matrix. The tensile direction is $[111]$, and a large number of stacking faults appear temporarily due to the movement of partial dislocations in Shockley. The stacking fault is funnel-shaped and consists of two "Pyramids" in relative positions. Satyajit Mojumder^27^ has studied the compression load effect of different Al crystal orientations on the plasticity of Al–Cu alloy by molecular dynamics method. For $\langle 001\rangle$ direction, the alloy can bear higher stress, while $\langle 110\rangle$ direction shows the opposite trend. For $\langle 111\rangle$ direction, the medium alloying degree has the highest strength. The elastic modulus in $\langle 111\rangle$ direction is the highest and the yield strain is the smallest, while the yield stress in $\langle 110\rangle$ direction is the highest and the yield strain is the largest. Hao Zhang^28^ has studied the effect of uniaxial tensile direction on the deformation mechanism of hexagonal closely packed (HCP) titanium crystals by molecular dynamics simulation. It is found that a series of "HCP → BCC → FCC" phase transitions occur when the tensile load is in the direction of $[\overline{2 }110]$; when the tensile load is in the direction of $[0\overline{1 }10]$, the slip of prismatic dislocations controls the deformation of the crystals; the prismatic dislocations can be dissociated into Shockley dislocations on the base, resulting in the formation of basal stacking faults. When the tensile load is in the direction of $[0001]$, a large number of $\langle c+a\rangle$ incomplete dislocations and Shockley partial dislocations are formed in the crystal, which is derived from the dissociation of $\langle c+a\rangle$ dislocations.

**Figure 1:**
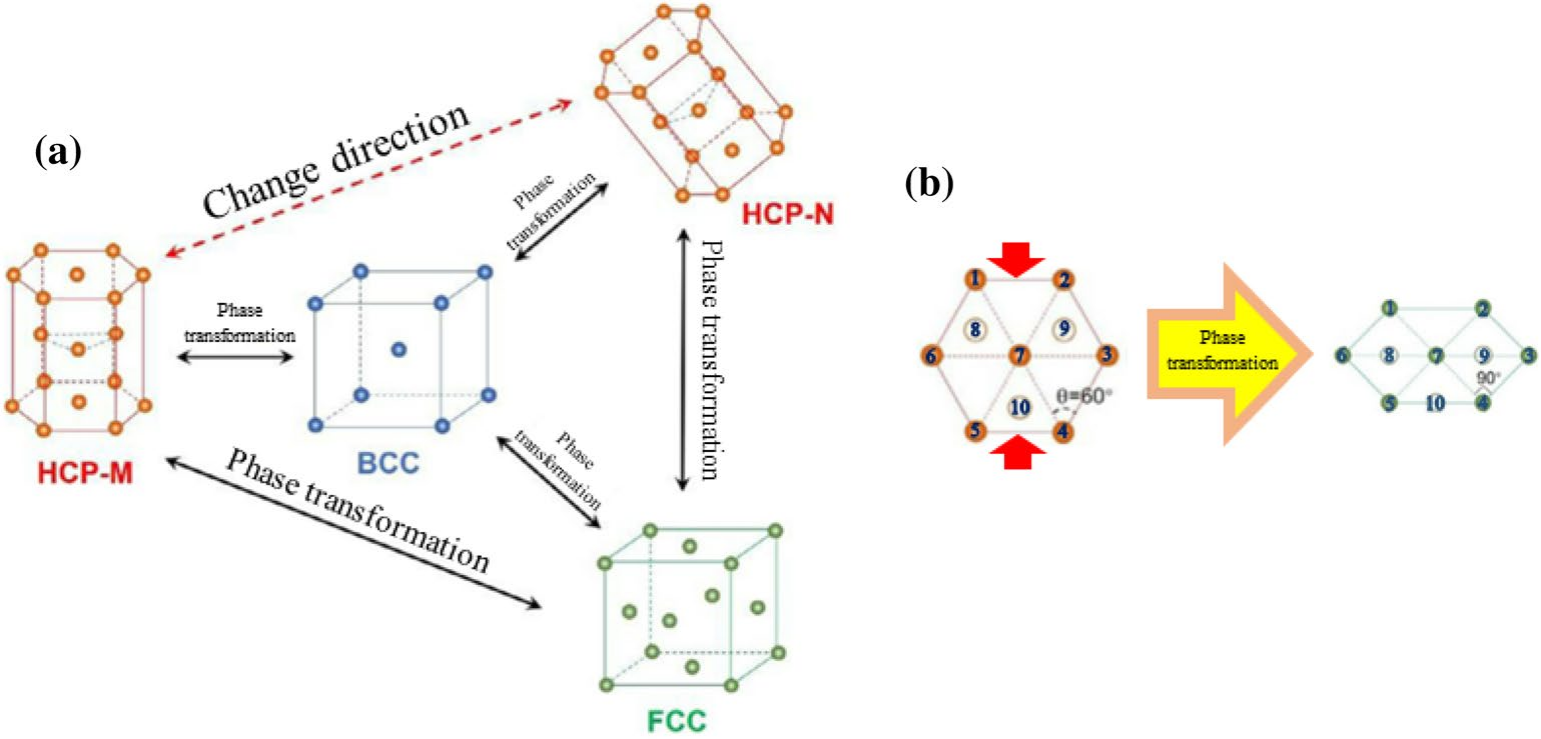
Phase transformation principle of AZ31 magnesium alloy.

Figure 1a shows the possible phase transition of AZ31 magnesium alloy during compression. When the magnesium alloy is compressed, the HCP structure of magnesium alloy changes and may turn from the original HCP-M phase to an angled HCP-N phase, or caused by the change of atomic position, one is to transform into BCC phase, the other is to transform into FCC phase. Similarly, the BCC phase or FCC phase can also form the HCP phase due to the change of atomic position. Figure 1b is the phase transition mechanism of HCP → FCC. It is found that when the HCP structure is compressed, the distance of atoms in the horizontal direction increases, and the distance in other directions shortens. The positions of atoms 8, 9, and 10, which were originally located in the interior of the HCP structure, do not change. However, due to the shortening of the distance between atoms 1 and 5, 2 and 4, atoms 8, 9, and 10 are already on the surface. The ∠347 originally is 60°, but now is 90°. At this time, the HCP structure becomes the FCC structure, and the FCC phase transition occurs.

To sum up, there is a lack of research on the relationship between phase transformation and loading direction of magnesium alloy. Therefore, to explore the effect of different loading directions on the phase transformation of magnesium alloy, the model of AZ31 magnesium alloy is established, the process of uniaxial compression (Uniaxial Compression, UC) of magnesium alloy along $\left[\overline{1 }2\overline{1 }0\right]$, $\left[\overline{1 }010\right]$ and $\left[0001\right]$ directions are simulated, the changes of atomic position and phase structure are observed. The phase transformation mechanism of AZ31 magnesium alloy under uniaxial compression under different loading directions is summarized. and the phase transformation mechanism of AZ31 magnesium alloy under different loading directions is summarized. It provides a theoretical basis for the development of new magnesium-based nano-alloys and an idea for new processing methods of magnesium alloys.

## Simulation means and methods

LAMMPS refers to a large-scale atomic/molecular massively parallel simulator, which can simulate the processing of nanomaterials^29^. The potential function is used to describe the relationship between the total energy $E$ of the model particles and the $(r)$ coordinates of the particles^30–32^. The embedded atomic potential (EAM) is usually used to calculate the interaction between metal and metal alloy atoms. The improved embedded atomic potential (MEAM) can be used to calculate the pairwise interactions of metals and alloys with FCC, BCC, HCP, and Diamond structures^33–39^. The total energy $E$ of the atomic system is shown in the formula:

$$ E = \sum\limits_{i} {\left\{ {F_{i} \left( {\overline{\rho }_{i} } \right) + \frac{1}{2}\sum\limits_{i \ne j} {\phi_{ij} \left( {\gamma_{ij} } \right)} } \right\}} . $$

In the formula, $E$ is the total energy of atomic $i$. $F$ is the embedded energy, which is a function of atomic electron density $\rho$. $\rho$ is atomic electron density. $\gamma$ and $\phi$ are a pair of potential interactions. And $i$ and $j$ are element types.

**Figure 2:**
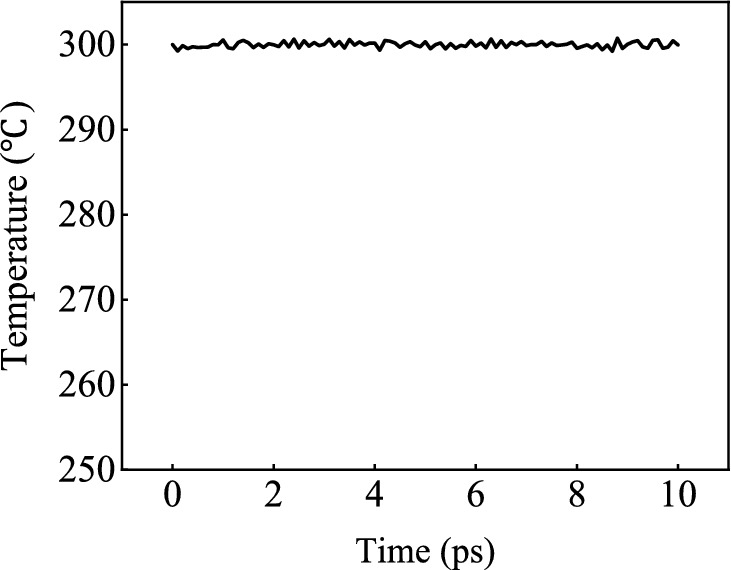
The MSD curve of relaxation temperature.

**Figure 3:**
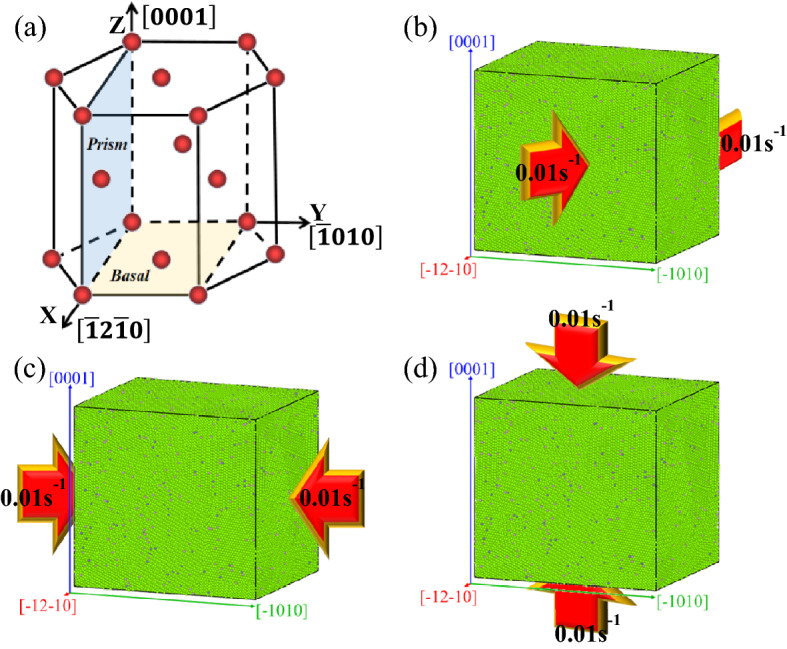
Diagram of the process. (**a**) Hexagonal closely packed cell; (**b**) compress along $\left[\overline{1 }2\overline{1 }0\right]$; (**c**) compress along $\left[\overline{1 }010\right]$; (**d**) compress along $\left[0001\right]$.

First of all, a Nanocrystalline Mg model with a size of 192.6 Å × 222.395 Å × 208.4 Å is established^40^. The number of atoms is 384,020, and the initial number of grains is 20. The X-axis, Y-axis, and Z-axis are set to correspond to the crystal directions of $\left[\overline{1 }2\overline{1 }0\right]$, $\left[\overline{1 }010\right]$ and $\left[0001\right]$, respectively. Then the AZ31 magnesium alloy nano-model is made by replacing 3% Mg atoms with Al atoms and 1% Mg atoms with Zn atoms. The established sample model is introduced into LAMMPS, and the Mg–Al–Zn potential function developed by Hyo-Sun Jang^41^ is assigned to the model. This potential function can be used to study the deformation and recrystallization properties of Mg–Al–Zn alloy in the whole processing temperature range. X, Y, and Z are all periodic boundary conditions, using the NPT ensemble. All simulations are performed using the Velocity-Verlet algorithm with a constant time step, and the ambient temperature is 300 K. First, to create a well-equilibrated sample, the model is initially relaxed to the minimum energy configuration by the conjugate gradient method. And the model is relaxed for 10 ps under constant temperature and pressure, The MSD curve of relaxation temperature is shown in Fig. 2. And then the strain rate of 0.01 ps^−1^ is uniformly compressed at 20 ps along the crystal direction $\left[\overline{1 }2\overline{1 }0\right]$, $\left[\overline{1 }010\right]$ and $\left[0001\right]$ respectively. Finally, the deformation of the magnesium alloy reaches 20%. The process diagram is shown in Fig. 3.

The simulation results are imported into the visualization software OVITO, and the functions: Common neighbor analysis (CNA), Dislocation analysis (DXA), Wigner–Seitz defect analysis (WSDA), and Construct surface mesh (CSM) are used for analysis. CNA provides visualization of common crystal structures in metals, such as face-centered cubic (FCC), body-centered cubic (BCC), hexagonal closely packed (HCP) structure, and Other structure (grain boundary atoms). DXA can determine the Burgers vector of each dislocation and identify the dislocation junction. WSDA can identify the crystal gap and calculate the number of interstitial atoms. CSM can construct a three-dimensional surface formed by a group of particles to observe the voids generated during the simulation^42–48^.

## Simulation results and analysis

**Figure 4:**
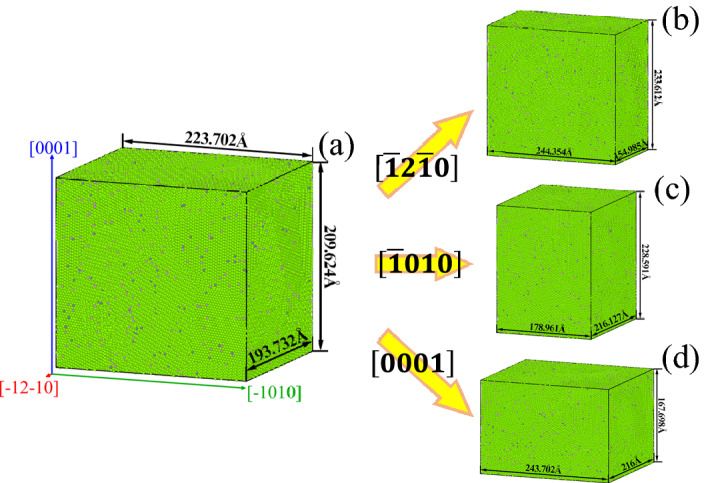
Compression result.

Figure 4a is an undeformed AZ31 magnesium alloy with a size of 193.732 Å × 223.702 Å × 209.624 Å. And Fig. 4b shows a magnesium alloy compressed 20% along $\left[\overline{1 }2\overline{1 }0\right]$, its size becomes 154.985 Å × 244.354 Å × 233.612 Å. Figure 4c shows a magnesium alloy compressed 20% along $\left[\overline{1 }010\right]$, and its size changes to 216.127 Å × 178.961 Å × 228.591 Å. Figure 4d shows a magnesium alloy compressed 20% along $\left[0001\right]$, and its size changes to 216 Å × 243.702 Å × 167.698 Å.

**Figure 5:**
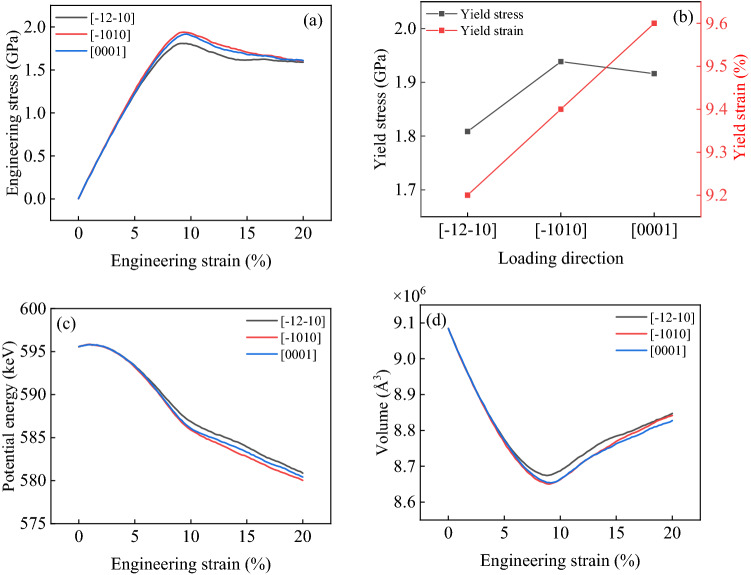
Performance curve. (**a**) Engineering stress–engineering strain curve; (**b**) yield stress–yield strain curve; (**c**) potential energy curve; (**d**) volume curve.

Figure 5a shows the engineering stress–engineering strain curve of AZ31 magnesium alloy under uniaxial compression in different directions, and Fig. 5b shows the Yield stress (maximum stress) and Yield strain (strain corresponding to Yield stress) of AZ31 magnesium alloy compressed in different directions. It is found that the minimum Yield stress is along $\left[\overline{1 }2\overline{1 }0\right]$ compression, the maximum Yield stress is along $\left[\overline{1 }010\right]$ compression, but the maximum Yield strain is along $\left[0001\right]$ compression. According to Fig. 5a, $\varepsilon <5\%$, the elastic modulus is the same when compressed in different directions. When $5\%<\varepsilon <UTS\%$, the elastic modulus changes^49,50^, and the elastic modulus is the highest when compressed along $\left[\overline{1 }010\right]$ and the lowest when compressed along $\left[\overline{1 }2\overline{1 }0\right]$.

Figure 5c shows the potential energy curve of AZ31 magnesium alloy compressed in different directions, and Fig. 5d shows the volume curve. It is found that $\varepsilon <5\%$, When compression along with different directions, the potential energy change is the same as the volume change. And the difference between potential energy change and volume change begins to appear after $\varepsilon >5\%$. This difference is consistent with the Ture stress-Ture strain curve. According to Fig. 5d, in the elastic stage, the volume of magnesium alloy decreases under compression, and after entering the plastic stage, the internal phase transformation of magnesium alloy occurs, forming FCC stacking fault (FCC SF), and the volume of magnesium alloy increases under compression.

**Figure 6:**
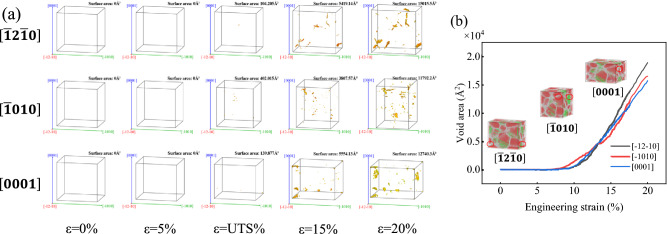
Evolution of voids.

Figure 6a is the schematic diagram of the evolution of voids in AZ31 magnesium alloy during compression in different directions. It can be found that voids are mostly formed at grain boundaries. When $\varepsilon >\mathrm{UTS}\%$, voids begin to form in magnesium alloys, and the number and area of voids increase with the increase of compression amount. Figure 6b shows the voids evolution curve of AZ31 magnesium alloy during compression in different directions. It is found that the voids evolution is consistent with the change of Ture stress–Ture strain curve. $\varepsilon <5\%$, with the increase of the amount of squeezing, the atomic position changes, and there are no voids in the model, and they are in the elastic state. $\varepsilon >5\%$, as the squeezing continues to increase and the atomic position changes, the atoms of the HCP structure become Other structure, and the number of grain boundaries increases and voids begins to appear in the model. When $\varepsilon >\mathrm{UTS}\%$, the Other structure becomes FCC SF, the void area increases sharply, the model volume increases, and the magnesium alloy enters the plastic stage.

**Figure 7:**
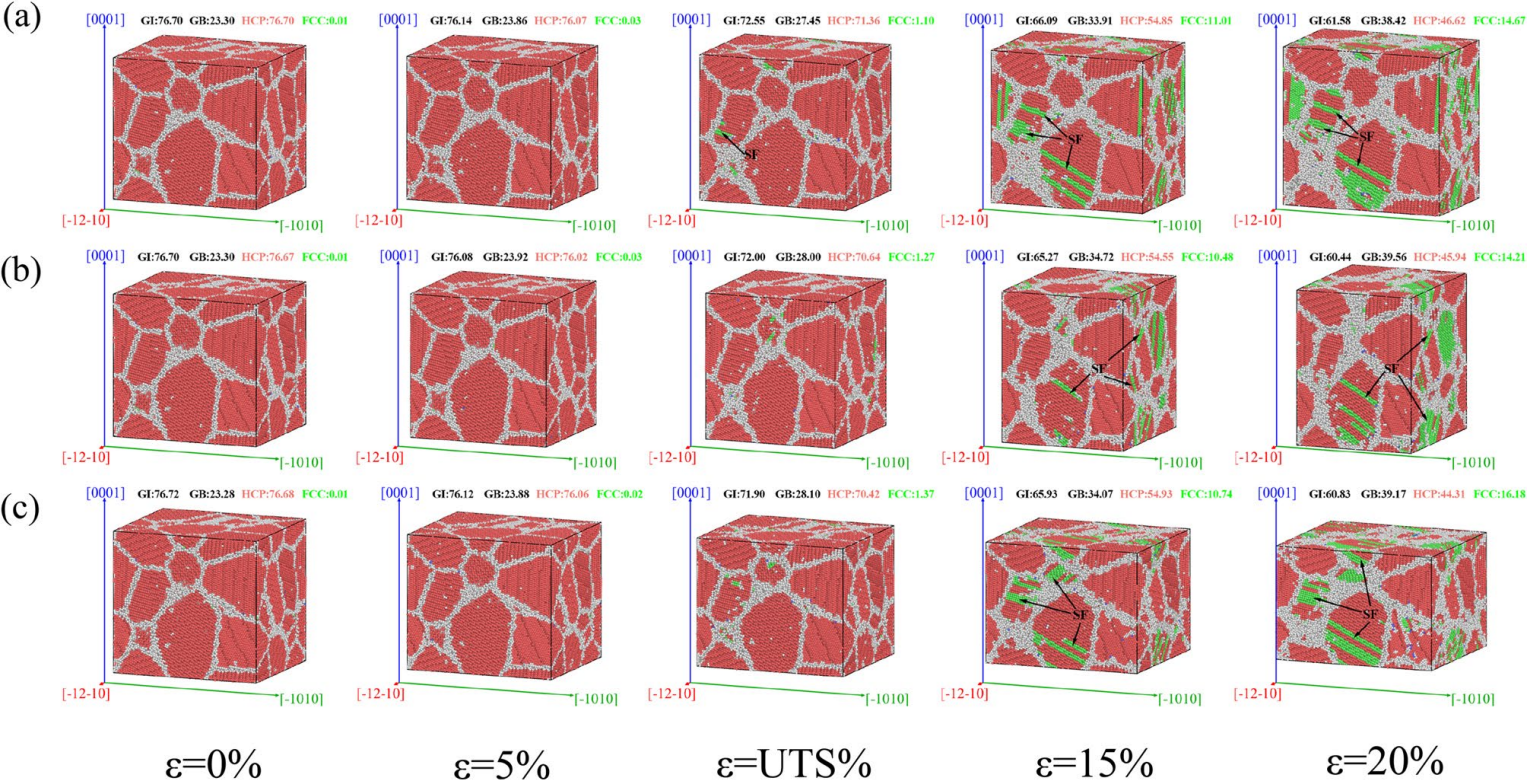
CNA diagram of AZ31 magnesium alloy during compression. (**a**) Compress along $\left[\overline{1 }2\overline{1 }0\right]$; (**b**) compress along $\left[\overline{1 }010\right]$; (**c**) compress along $\left[0001\right]$.

Figure 7 shows the CNA diagram of AZ31 magnesium alloy when it is compressed in different directions. The atoms in the picture have been colored according to CNA. The red atoms are HCP structure, the green atoms are FCC structure, and the white atoms are Other structure (that is made up the grain boundary, GB atoms). According to Fig. 7, it is found that as compression progresses, the atomic position changes, and so does the phase structure. After compression, the position of the atoms of the HCP structure changes to the atoms of the Other structure, forming the grain boundary. As the compression continues, the positions of some atoms continue to change, and the Other structure changes into the FCC structure, forming FCC SF. When $\varepsilon >\mathrm{UTS}\%$, the FCC SF increases rapidly. When magnesium alloy is compressed, its structure evolves to HCP → Other → FCC.

**Figure 8:**
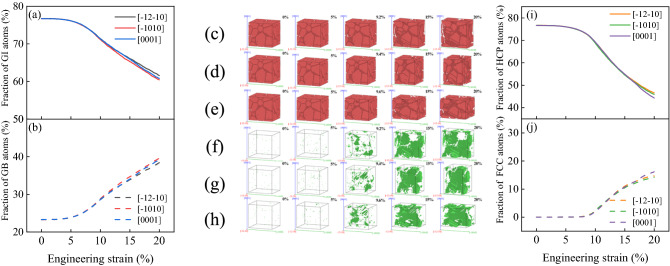
Phase structure change curve during compression. (**a**) Fractional curve of GI atoms; (**b**) fractional curve of GB atoms; (**c**) ~ (**e**) HCP structure evolution; (**f**) ~ (**h**) FCC structure evolution; (**i**) fractional curve of HCP structure atoms; (**j**) fractional curve of FCC structure atoms.

Figure 8 shows the phase structure change curve of AZ31 magnesium alloy during compression in different directions. Figure 8a shows the fractional curve of GI atoms, Fig. 8b shows t the fractional curve of GB atoms, Fig. 8c–e shows the evolution of HCP structure, Fig. 8f–h shows the evolution of FCC structure, Fig. 8i shows the fractional curve of HCP structure atoms, and Fig. 8j shows the fractional curve of FCC structure atoms. According to Fig. 8a,b, we can see that $\varepsilon <5\%$, with the increase of the amount of compression, the atomic fraction does not change, because there are no voids in the model and are in the elastic state. $\varepsilon >5\%$, as the amount of compression, continues to increase, the GI atoms become GB atoms, the number of grain boundaries increases, and voids begin to appear in the model. After $\varepsilon >\mathrm{UTS}\%$, the void area increases sharply, the model volume increases, and the magnesium alloy enters the plastic state. According to Fig. 8c–j, we can see that the atoms of HCP structure decrease rapidly when $\varepsilon =5\sim 20\%$, while the atoms of FCC structure increase sharply when $\varepsilon =\mathrm{UTS}\%\sim 20\%$, and the HCP transition FCC has a transition stage of about 4.5%. At this stage, the HCP structure first changes into the Other structure, and when $\varepsilon >\mathrm{UTS}\%$, the Other structure becomes the FCC structure. The change of phase structure of magnesium alloy is the same when it is compressed along with all directions, and there is no obvious difference. Therefore, in the process of uniaxial compression of magnesium alloy, the phase transformation of HCP → FCC is the main one.

**Figure 9:**
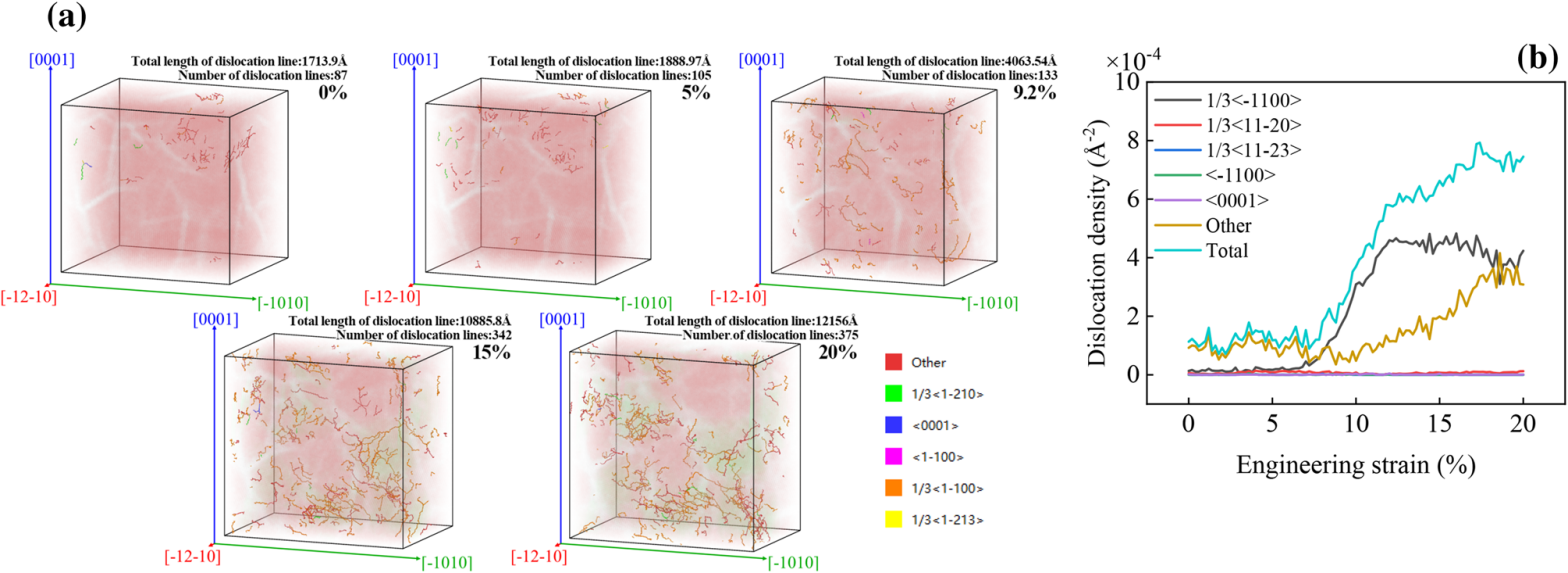
Dislocation evolution and density curve during compression along $\left[\overline{1 }2\overline{1 }0\right]$.

Figure 9 shows the dislocation evolution and density curve of AZ31 magnesium alloy during compression along $\left[\overline{1 }2\overline{1 }0\right]$, Fig. 9a shows the dislocation evolution in the model, the atoms in the figure have been treated transparently, and Fig. 9b shows the dislocation density curve. According to Fig. 9a, it can be found that the most obvious stage of dislocation growth is $\varepsilon =5\sim 20\%$. As the number of compression increases, the atomic position changes, and the number and length of dislocations increase. According to Fig. 9b, it can be found that $\varepsilon <5\%$, with the increase of compression, the dislocations in the model are mainly Other dislocations, that is unrecognizable dislocations. At this time, what happens in the model is the transition of HCP → Other, which is in an elastic state. $\varepsilon >5\%$, with the increase of compression amount, there are $1/3<\overline{1 }100>$ dislocations(Shockley partial dislocations) in the model, Other structure increases, and the number of grain boundaries increases. When $\varepsilon >\mathrm{UTS}\%$, part of the Other structure becomes FCC SF, and the magnesium alloy enters the plastic state. According to Fig. 8, the dislocation evolution of magnesium alloy during compression in all directions is consistent, and there is no significant difference in dislocation density.

**Figure 10:**
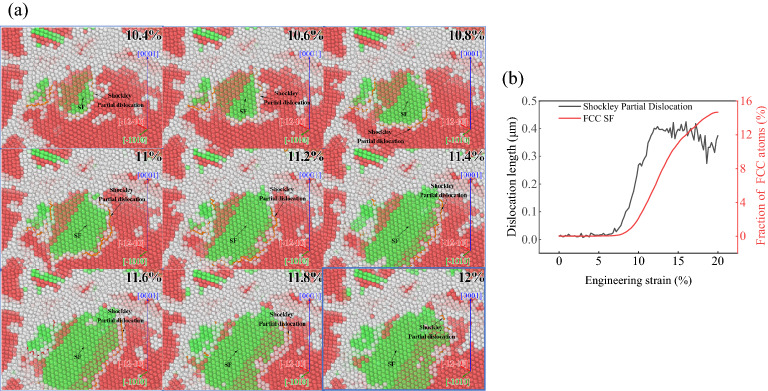
Correlation between Shockley partial dislocations and FCC SF.

Figure 10a shows the growth of Shockley partial dislocations and FCC SF of AZ31 magnesium alloy during compression along $\left[\overline{1 }2\overline{1 }0\right]$. To observe the positions of dislocations and SF, the atoms are treated transparently, and the positions of Shockley partial dislocations and FCC SF are marked. It can be found that Shockley partial dislocations always surround FCC SF. With the increase of the amount of compression, the atoms of HCP structure first become Other structure, resulting in Shockley partial dislocations, the atomic position changes continuously, the dislocations move, and the atoms of Other structure become FCC structure, forming FCC SF.

Figure 10b shows the curves of the growth of Shockley partial dislocations and FCC SF under different compression directions. It is found that Shockley partial dislocations always occur about 4.5% earlier than FCC SF. This is because the atomic position changes during compression, but does not directly change from HCP phase structure to FCC phase structure, but slips first to become Other structure atoms, resulting in Shockley partial dislocations. With the increase of compression, Shockley partial dislocations increase. When $\varepsilon >\mathrm{UTS}\%$, the FCC SF is formed and the volume increases.

## Conclusion

In this paper, to study the effect of loading direction on uniaxial compression of AZ31 magnesium alloy, the model of AZ31 magnesium alloy was established. The uniaxial compression process of magnesium alloy along $\left[\overline{1 }2\overline{1 }0\right]$, $\left[\overline{1 }010\right]$ and $\left[0001\right]$ was simulated by molecular dynamics, the changes of atomic position and phase structure were observed, and the phase transformation mechanism of AZ31 magnesium alloy under different loading directions was summarized. The main results are as follows:

1. Engineering stress–engineering strain, potential energy and volume change, void evolution, phase structure change, and dislocation evolution of magnesium alloy are consistent with each direction of compression, and there is no obvious difference.
2. $\varepsilon <5\%$, the atomic position changes with the increase of compression, the dislocations in the model are mainly Other dislocations, that is unrecognizable dislocations, what happens at this time is the transition of HCP structure → Other structure. There are no voids in the model and are in an elastic state. $\varepsilon >5\%$ The atom of the HCP structure becomes Other structure. $1/3<\overline{1 }100>$ dislocations are generated in the model (Shockley partial dislocations). And the number of grain boundaries increases, and the voids begin to appear at this time. $\varepsilon >\mathrm{UTS}\%$, the Other structure becomes FCC SF. the void area increases sharply, the model volume increases, and the magnesium alloy enters the plastic state.
3. In the process of uniaxial compression of magnesium alloy, the phase transformation of HCP → FCC is the main. HCP → FCC has a transition stage of about 4.5%, and its structure evolves into HCP → Other → FCC. The atoms of the HCP structure are compressed and become the atoms of the Other structure, forming the grain boundary. With the continuation of the compression, the Other structure becomes the FCC structure, forming the FCC SF. When $\varepsilon >\mathrm{UTS}\%$, the FCC SF increases rapidly.
4. Shockley partial dislocations always occur about 4.5% before FCC SF, and Shockley partial dislocations surround FCC SF.

## Data Availability

The datasets generated during and/or analysed during the current study are available from the corresponding author on reasonable request.
